# A Dictionary of Practical Surgery; Comprehending All the Most Interesting Improvements up to the Present Period. The Third Edition, Revised, Corrected, and Enlarged

**Published:** 1818-03

**Authors:** 


*A Dictionary of Practical Surgery; comprehending all the most interesting Improvements up to the present Period. The* Third *Edition, revised, corrected, and enlarged.* By SAMUEL COOPER, one of the Surgeons to His Majesty's Forces, Member of the Royal College of Surgeons, &c. Octavo, pp. 1122. London, 1818.

WE confess we are somewhat jealous of Encyclopedias, Dictionaries, and Compendiums, seeing our minds are by no means made up, whether to regard the multiplication of such publications as tests of the growth or decay—the health or decline of real science. Of one thing, however, we are pretty certain, namely, that those who purchase these expensive publications, generally pay a most extravagant price for their information, since, along with the subjects in which they feel an interest, they necessarily buy a farrago of other matter, which, to them, is too often totally useless. The success of the sale, therefore, instead of indicating a general taste for science, may be pretty generally regarded as a manifestation of the powerful principle of acquisition in the human mind, which prompts us to appropriate to ourselves that of which, in all likelihood, we shall make no use.

Besides, the young or frivolous portion of mankind generally have recourse to such works as *short cuts* to knowledge. In such hands, their too frequent effect is to engender speciousness and petulance, to paralyse the spirit of research, and to substitute the froth and scum of science in lieu of its healthful and pellucid waters. Hence, so many are taught "to talk like angels," while, in reality, their knowledge is inaccurate and unsubstantial; and use a familiar freedom with books (as some silly persons do with Lords), of which they know only the names or the titles.

But, without entering farther into the abstract question as to the general utility of such publications, it must be evident, that on any *single* science, such as ours, a compendious concentration of the scattered knowledge of its innumerable professors and writers must be highly valuable. Nay, we contend, that it is indispensably necessary; for, since no medical man of ordinary application or leisure, can, now-a-days, pretend to read all the works that emanate from our professional press, the duty of judicious selection and condensation becomes a very requisite,—and, if well executed,—a very honourable one.

Of the truth of these remarks, the success which has attended the two former editions of this work is a conspicuous proof. Those who are engaged in the hurry of actual practice, who have sustained its anxiety and its responsibility, and have felt how much we would give, now and then, for any light, however feeble, either from men or books, will not fail to set a just value on a compendium like the present, which embraces every material point, both of surgical pathology and practice. It will be peculiarly acceptable to practitioners in the country, who from their locality, and their long absence from the schools of the metropolis, are apt to be left behind in the rapid marches of our art. Of its recent progress, and its actual state at this hour, we know of no more faithful summary than the Dictionary of Mr. Samuel Cooper.

The nature of the work necessarily precludes even the most distant attempt at analysis; indeed, the latter would be improper, as we would wish the book itself to find its way into every library: besides, it has been already placed beyond the reach of criticism by the great seal of public favour. But, for the sake of those who are possessed of the former editions, we shall give a summary of the new matter set forth in the preface to the present.

Most of our readers are aware, that the justly distinguished Mr. Astley Cooper, some years ago, tied the abdominal aorta in a dog; and that the circulation was still carried on by the arteries both on the fore and back part of the trunk. The beautiful dilatation of these vessels formed an interesting preparation, when the animal was, some time afterwards, put to death. This enterprising gentleman has lately performed a similar operation in Guy's Hospital, on the human subject.

"The patient had an aneurism in the groin, extending so far within the body, that the operation of taking up the external iliac artery appeared totally impracticable. The tumour had already burst, and the patient was on the point of losing his life by hæmorrhage. In this dilemma, the above celebrated surgeon, ever anxious to enlarge the limits of chirurgical knowledge, determined first to try to get at the source of the bleeding, by opening the swelling; and if nothing could be done in this manner, he resolved to make his way to the aorta, by an incision through the parietes of the abdomen, and then to apply a ligature to this trunk of trunks. Suffice it here to state, that the aorta was tied; that immediate death from hæmorrhage was prevented; and, what is above all things interesting, that the circulation went on in the lower extremities. The leg, however, on the same side as the tumour, became cold; but the opposite one retained its natural warmth. The man lived thirty-six hours after the operation.

"With the particulars, however, respecting the symptoms preceding his death, the exact part of the artery tied, and the appearances on dissection, I am not, at present, acquainted."*— Preface, p. 4.

It also appears, that the internal iliac artery has been recently tied for a glutæal aneurism, by Mr. Atkinson of York. The patient, however, sank on the 19th day from the operation, partly in consequence of the profuseness of the discharge, and partly in consequence of hæmorrhage.

We conclude with the following remark on the diminished mortality after the operation for lithotomy.

"I think nearly half the patients, formerly operated on in St. Bartholomew's Hospital, had peritonitis afterwards; but, at present, the deaths are so diminished, that not a single patient has died there after lithotomy for more than two years past; and peritonitis is a much less common consequence of the operation. Now, although it must be acknowledged that lithotomy has not been done so often as usual at St. Bartholomew's during the last year or two; yet, the fact of no patients having been lost, seems to evince some improvement in the instruments employed, or in the mode of operating. At all events, it should be known, that beaked knives have there been very commonly substituted for gorgets, which, a few years ago, were really terrific instruments, partly on account of their bad construction, and partly by reason of the mischief, which often arose from their slipping off the staff." Preface, p. 16.

For these extracts, we trust, we need make no apology to our readers.

---

*Observations on some Important Points in the Practice of Military Surgery, and in the Arrangement and Police of Hospitals; Illustrated by Dissections.* By JOHN HENNEN, Deputy Inspector of Hospitals. pp. 520. *Edinburgh, London, Dublin.* 1818.

"LES circonstances," says a recent French writer on mi-

---

* The whole of this interesting and melancholy Case will be found in a subsequent part of our present Number. EDIT.

